# Host-microbe interactions in octocoral holobionts - recent advances and perspectives

**DOI:** 10.1186/s40168-018-0431-6

**Published:** 2018-04-02

**Authors:** Jeroen A. J. M. van de Water, Denis Allemand, Christine Ferrier-Pagès

**Affiliations:** Centre Scientifique de Monaco, 8 Quai Antoine 1er, 98000 Monaco, Monaco

**Keywords:** Microbiome, Holobiont, *Symbiodinium*, Immunity, Bacteria, Fungi, Gorgonians, Octocoral, Soft coral

## Abstract

Octocorals are one of the most ubiquitous benthic organisms in marine ecosystems from the shallow tropics to the Antarctic deep sea, providing habitat for numerous organisms as well as ecosystem services for humans. In contrast to the holobionts of reef-building scleractinian corals, the holobionts of octocorals have received relatively little attention, despite the devastating effects of disease outbreaks on many populations. Recent advances have shown that octocorals possess remarkably stable bacterial communities on geographical and temporal scales as well as under environmental stress. This may be the result of their high capacity to regulate their microbiome through the production of antimicrobial and quorum-sensing interfering compounds. Despite decades of research relating to octocoral-microbe interactions, a synthesis of this expanding field has not been conducted to date. We therefore provide an urgently needed review on our current knowledge about octocoral holobionts. Specifically, we briefly introduce the ecological role of octocorals and the concept of holobiont before providing detailed overviews of (I) the symbiosis between octocorals and the algal symbiont *Symbiodinium*; (II) the main fungal, viral, and bacterial taxa associated with octocorals; (III) the dominance of the microbial assemblages by a few microbial species, the stability of these associations, and their evolutionary history with the host organism; (IV) octocoral diseases; (V) how octocorals use their immune system to fight pathogens; (VI) microbiome regulation by the octocoral and its associated microbes; and (VII) the discovery of natural products with microbiome regulatory activities. Finally, we present our perspectives on how the field of octocoral research should move forward, and the recognition that these organisms may be suitable model organisms to study coral-microbe symbioses.

## Background

**Fig. 1:**
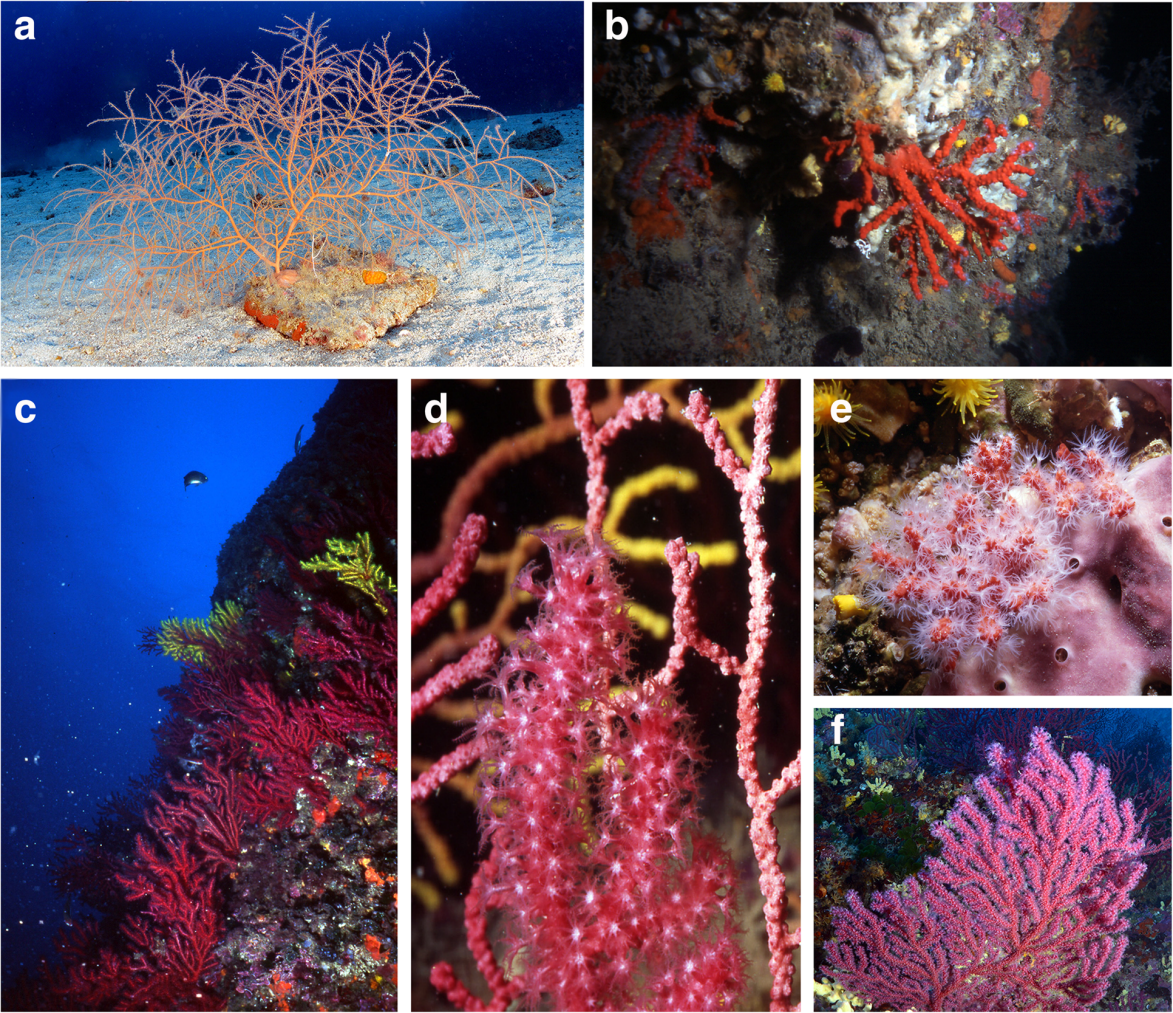
Octocorals as habitat providers. **a**–**c** Gorgonians form three-dimensional structures in a range of environments, such as **a** *Leptogorgia sarmentosa* on sandy bottoms, **b** *Corallium rubrum* on the walls and ceilings of caves and overhangs, and **c** *Paramuricea clavata* forming “marine animal forests” on rocky substrates. Close-up of the gorgonian colonies of **d** *Paramuricea clavata* with open (front) and retracted (back) polyps and **e** *C. rubrum*, showing their eightfold body symmetry. **f** Colonies can consist of thousands of polyps forming large three-dimensional structures. Together colonies can form vast “forests” providing refuge and habitat for numerous marine organisms (photos **a** and **f** by Eric Béraud and photos **b**–**e** by Sergio Rossi)

The Octocorallia (Haeckel, 1866) is a subclass within the Anthozoans (Ehrenberg, 1834, phylum Cnidaria (Verrill, 1865)) and is comprised of soft corals, including sea fans and sea whips (order Alcyonacea (Lamouroux, 1812)), sea pens (order Pennatulacea (Verrill, 1865)), and blue corals (order Helioporacea (Bock, 1938)). The main characteristic of Octocorallia that distinguishes them from the Hexacorallia (Haeckel, 1896), such as the reef-building Scleractinia (Bourne, 1900), is the eightfold symmetry of their polyps (Fig. 1), compared to the sixfold symmetry in their relatives. To date, over 3500 species belonging to approximately 378 octocoral genera from 55 families [1, 2] have been described worldwide. Some of those have been famous since the Classical Antiquity: for example, the beautifully red skeleton of the precious red coral *Corallium rubrum* (Fig. 1) has been extensively used for jewelry and other art crafts [3]. Octocorals are ubiquitous organisms of the sea, having been recorded at all depths, from littoral waters down to the deep-sea abyss, from the tropics to the arctic regions, and in all the world’s oceans, although the highest diversity of octocorals is observed in the Indo-Pacific (reviewed in [4]). While octocoral distribution is significantly influenced by various environmental factors [5], the presence of octocorals in nearly all benthic marine habitats indicates the adaptive nature of this taxonomic group compared to other cnidarian taxa. In some geographical areas, reef ecosystems have even undergone a phase shift from a hard coral-dominated state towards a higher abundance of soft corals (Table 1).

**Table 1:** Reported coral community shifts from reef-building scleractinian corals to soft corals and their causes

Location		Cause	Shift in octocoral cover		References
			From (%)	Up to (%)	
Red Sea	North and Center	Crown of thorns starfish Anthropogenic pollution	5–10	30–50	[245–247]
	South	Storms, bleaching Anthropogenic pollution	4	30	[248, 249]
Caribbean	Florida Keys	Bleaching	6	14	[159]
	US Virgin Islands	Diseases			[250]
Indian Ocean	Madagascar/Seychelles	Bleaching	1.3	2	[251]
	Seychelles	Bleaching and anthropogenic pollution			[245]
Pacific Ocean	Malaysia	Bleaching Dynamite fishing Crown of thorns starfish	1	30	[252]
	Indonesia	Anthropogenic pollution Dynamite fishing	7	13	[253, 254]
	Australia	Bleaching Crown of thorns starfish	10	16	[255]
	Fiji	Anthropogenic pollution	N/A	N/A	[256]

Octocorals are important foundational members of the benthic community. Through the formation of three-dimensional structures, they provide structural complexity to ecosystems and thereby refuge and habitats to a rich fauna (Fig. 1). While all octocorals are suspension feeders, relying upon currents to have access to food (reviewed in [6]), some octocorals also live in a mutualistic association with phototrophic zooxanthellae (dinoflagellates from the genus *Symbiodinium*). Although these zooxanthellate octocorals are restricted to the euphotic zone, they significantly contribute to the primary productivity of the shallow coastal ecosystems [7]. Azooxanthellate octocorals, however, rely solely on heterotrophic feeding and generally populate dark and deep environments, where they can develop very dense populations providing biomass and structural complexity [8, 9]. Because of their abundance, octocorals play a major role in the benthic-pelagic coupling and the energy transfer between plankton and benthos as they capture large quantities of plankton and thereby regulate the primary and secondary productions of the coastal food chains [6].

As all multicellular organisms, corals (encompassing both the Octocorallia and Hexacorallia) are holobiont entities, forming intricate and complex interactions with a range of microbes, including dinoflagellates, fungi, bacteria, archaea, and viruses [10]. These microbial symbionts play active roles in the health (e.g., nutrient supply, protection against pathogens) and adaptive response (e.g., toxin degradation) of the host to environmental changes [11, 12]. In zooxanthellate corals, endosymbiotic photosynthetic dinoflagellates (*Symbiodinium*) are the main food providers to their coral host, via the transfer of carbon rich compounds acquired through photosynthesis, as well as the recycling of nitrogen and phosphorus through the host catabolic wastes [13]. In corals, bacterial symbionts have been implicated in several other services, such as nitrogen fixation [14], sulfur cycling [15], or antibiotic production to exclude pathogens [16]. Maintaining a multi-functional microbial community is therefore essential to holobiont fitness. Recent studies have shown that corals have a “core microbiome” [17], composed of microbes that are consistently associated with a host species, as well as transient microbes whose presence depends on local conditions [18–20]. In case of environmental stress, such as rising sea water temperatures, changes in the resident microbial community composition and function may occur and lead to the occurrence of transient pathogens and to the emergence of disease [21]. Although the composition of the coral microbiota has been studied extensively under a range of environmental and experimental conditions, the diverse functions of the bacteria within the coral holobiont are still largely unknown.

The majority of scientific studies and reviews on the subject has focused on the reef-building scleractinian corals [21–23] and has shown that the holobiont is a community of Dinoflagellata, bacteria, fungi, Archaea, and viruses. Octocoral-microbe interactions have comparatively received relatively little attention, with only a limited number of studies having addressed their associated fungal and bacterial communities. In addition, most studies have used culture-based methods, which are relatively limited as only few microbes are cultivable, before next-generation sequencing techniques became more affordable in recent years, allowing higher resolution of the composition of octocoral-associated microbial community. Because of the significant advances made in the octocoral microbiome field in recent years and the relevance of these findings for our general understanding on the structure, function, and evolution of coral-microbe symbioses, a comprehensive assessment is warranted.

In this review, we outline the recent discoveries and current knowledge regarding (I) the octocoral-*Symbiodinium* mutualism, (II) the diversity and function of microbes (including fungi, viruses and bacteria) associated with tropical, temperate, and cold-water octocorals, and (III) the structure and stability of the microbial assemblages and remarkable dominance of a few bacterial species that suggest a close evolutionary history. We will also address (IV) the potential for microbiome regulation by the host and (V) the octocoral immune system in case of (VI) the occurrence of infections and diseases. Lastly, we will discuss (VII) the potential application of natural products derived from octocoral holobionts. The aims of this review were to summarize the latest achievements and to highlight future research directions to build a mechanistic understanding of how coral health is connected through microbial processes to its surrounding environment.

## The algal symbiont *Symbiodinium*

Many octocoral species live in mutualistic association with unicellular algae (dinoflagellate of the genus *Symbiodinium*), also commonly called zooxanthellae. It is well-known now that these coral symbionts translocate carbon-based photosynthates to satisfy their host’s nutritional needs [13] as well as other nutrients such as nitrogen and phosphorus acquired from seawater or recycled from the host catabolic wastes [24]. By coupling this autotrophic nutrition, with the opportunistic heterotrophic feeding of the host (prey capture), many corals can thrive in nutrient-poor environments, also called oceanic deserts [25].

**Fig. 2:**
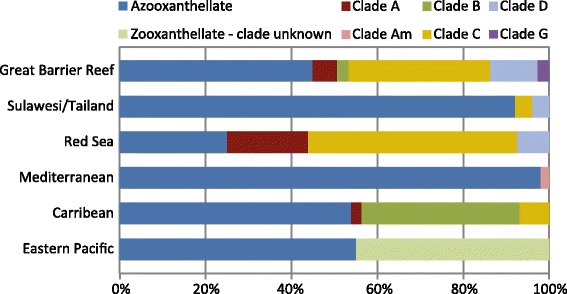
Relative abundance of octocorals harboring specific clades of *Symbiodinium* in different geographical areas. A large number of octocoral species is azooxanthellate and does not possess algal symbionts

In octocorals, *Symbiodinium* can be acquired via vertical transmission (maternal inheritance) or horizontal transmission (environmental acquisition) [26], although the incidence of vertical transmission seems to be higher than for scleractinians [27]. Octocorals harbor five of the nine distinct phylogenetic *Symbiodinium* clades (called A to I) known to live in symbiosis with different organisms [28] (Fig. 2). Generally, the diversity of octocoral-associated *Symbiodinium* is higher in the tropics compared to temperate regions [29, 30] and the highest diversity is found on the Great Barrier Reef [29, 31]. The majority of octocorals investigated so far, however, harbored only a single algal clade, showing geographical clustering patterns based on the dominant *Symbiodinium* types (Fig. 2). For example, Clade C is dominant in the Pacific Ocean and the Red Sea [29, 30], whereas Caribbean octocorals are dominated by Clade B [30, 32] and Mediterranean octocorals by the temperate Clade A [26]. Interestingly, these associations are also rather stable over time and space compared to scleractinian corals, even after thermal stress and bleaching [28, 33–35]. The stability of these interactions may be due to parental effects in the establishment of this mutualistic symbiosis. For example, offspring of *Briareum asbestinum* has been found to contain several symbiont types early on in the symbiosis, but ultimately engaged in a mutualistic relationship with the *Symbiodinium* phylotype that was also dominant in the parental colonies [36]. As this specificity was observed regardless of the environmental conditions, it brings into question the adaptability of the octocoral-*Symbiodinium* mutualism under changing climate conditions [36]. The prominent zooxanthella genotype may thus also exclude other genotypes attempting to enter the association, possibly through faster growth rates [37], or higher services provided to their host. Multiple strains of each clade of *Symbiodinium* were, however, found within a single octocoral host [29, 38, 39], with a high specificity between host and symbiont lineages [39] and a high degree of connectivity between *Symbiodinium* populations [40].

The octocoral-*Symbiodinium* association has received far less attention than those established with scleractinian corals. Therefore, there are critical gaps in our understanding of the functional and ecological significance of these symbioses. For example, the nutritional exchanges between the two partners, the trophic contribution of the symbionts to the energetic requirements of their host, and the stability of the symbiosis during environmental stress are still poorly understood in octocorals and deserve future attention.

Due to the paucity of zooxanthellate octocorals (compared to the azooxanthellate) both in the Indo-Pacific Ocean and temperate seas [29, 41] (Fig. 2), there is an impression that octocorals are heterotrophic species, relying on plankton and detrital material for their basic metabolism, growth and reproduction. However, there are more than 51 zooxanthellate octocoral species in the Caribbean [42], and zooxanthellate species also dominate the octocorals of the Southern Red Sea [43], suggesting that, in some locations at least, the association of octocorals with *Symbiodinium* can be mutualistic, providing a nutritional advantage. Productivity of zooxanthellate corals is usually estimated via the photosynthesis:respiration (P:R) ratio calculated over a daily cycle. A P:R > 1 indicates that the holobiont acquires more photosynthetic organic material than it consumes and can therefore rely on autotrophy for its energetic needs. The first few studies which have assessed the rates of photosynthesis and respiration of zooxanthellate octocorals measured very low rates of primary productivity compared to scleractinian corals, both in the temperate [44] and tropical [7, 45] areas. It was also observed that the presence of *Symbiodinium* increased metabolic costs, and thereby respiration rates [46], indeed suggesting that these octocorals have to rely on both auto-and heterotrophy to sustain their metabolic needs. Subsequent studies, performed on a greater number of species, however, showed that octocoral primary productivity depends on the environment, the polyp activity, the *Symbiodinium* clade identity, and the host morphology. For example, the P:R ratio tends to be the lowest in summer for tropical species (due to photoinhibition of the symbionts’ activity) [47], while it is the highest in summer for temperate areas, which are light limited during the other seasons [44, 48, 49]. For the same environment, the P:R ratio is also linked to the surface area:volume ratio (SA:V ratio). For example, sea fans (e.g., *Gorgonia ventalina*) are the most autotrophic octocorals due to their broad leaf-like morphology and small polyps (i.e., a high SA:V ratio), allowing efficient light exposure to the zooxanthellae and therefore maximized photosynthesis [50]. On the contrary, massive octocorals, with big polyps (e.g., *Plexaurella fusifera* and other sea rod species), are more suited to capturing plankton and particulate organic matter. Isotopic experiments with ^13^C-labeled inorganic carbon indeed showed that such big polyp hosts do not benefit from *Symbiodinium* autotrophy, due to low photosynthate translocation rates by these symbionts. As such, the host-*Symbiodinium* relationship in these octocorals is more commensal than mutualistic, at least for carbon, as previously observed in other host-microbe symbioses [51]. Overall, the negative phenotypic correlation observed between polyp size and carbon translocated from zooxanthellae to host suggests that there is an evolutionary trade-off between heterotrophic and autotrophic modes of nutrition. Finally, Baker et al. [50] found evidence that *Symbiodinium* specificity increases with holobiont productivity: generalist hosts (host with different symbionts) had lower productivities than specialist hosts (host with a specific symbiont type). Even if symbionts do not supply carbon compounds to their octocoral host, they can still be important for the acquisition of other essential nutrients such as nitrogen and phosphorus [45, 52].

As for all corals hosting algal symbionts, thermal stress (abnormally cold or warm temperatures) may lead to bleaching, i.e., the expulsion of the zooxanthellae, often due to an overproduction of reactive oxygen species (ROS) and increased oxidative stress [53, 54]. For example, high thermal anomalies induced extensive bleaching and mortalities of octocorals in the Pacific and the Caribbean in 1998, but also in 2005 and 2010 in the Florida Keys and wider Caribbean [53, 55]. The loss of zooxanthellae induces host starvation, when the host actively relies on photosynthates to sustain its metabolism. As such, it has been observed that species with large polyps, and/or a facultative symbiosis, whose nutrition is not derived exclusively from the symbionts, will bleach more easily than species forming an obligate association with their symbionts and receiving a large amount of photosynthates [50]. In the Caribbean, as many species have an obligate association with their symbionts, they were shown to be more resistant to temperature-induced bleaching than their scleractinian counterparts (reviewed in [56]). Instead of bleaching, symbiont migration into the stolon has been observed in some octocoral species, however, with a significant increase in ROS and an impairment of the photosynthesis [57–59]. Nevertheless, despite these physiological perturbations, symbionts retained some capacity for photosynthesis even after completing migration into the stolons.

## Fungi

Despite the impacts of fungal disease on gorgonian populations [60–62], relatively few studies have investigated the fungal community associated with soft corals. Identification and characterization of fungal isolates have shown the consistent associations of various fungal species and genera with octocorals around the world. Particularly, *Aspergillus* spp. and *Penicillium* spp. have been commonly isolated from *Gorgonia ventalina* in the Caribbean [63, 64], *Leptogorgia* spp. in the Eastern Pacific [65], and numerous octocorals in Singapore [66] and the South China Sea [67]. Other common fungal associates of octocorals belong to genera *Cladosporium* [63–66], *Tritirachium* [63–66], *Nigrospora* [65, 67], and *Fusarium* [65–67]. Local environmental conditions, however, appeared to affect fungal community compositions [63, 67], primarly showing differences in the abundances of the most common fungal associates. While the functional ecological roles of these fungi are unknown, some possess potent antibacterial and/or antifungal activity and have been suggested to play a role in holobiont health and microbiome regulation [67]. One of the most notorious fungi, the putative aspergillosis pathogen *Aspergillus sydowii*, was found on both healthy and diseased gorgonians, although it was absent in some diseased colonies [63, 64]. As such, this fungus may in fact be an opportunist rather than a primary disease-causing pathogen. *A. sydowii* as well as nearly all other fungal isolates were initially considered terrestrial microbes, and some data suggests that these fungi could be derived from terrestrial run-off as (1) the host-associated fungal community does not differ from the seawater community and (2) the offshore fungal seawater communities resemble a diluted nearshore community [63]. One of the limitations in studies on octocoral-associated fungi has been the use of culture-based techniques, as the culture media used have been shown to be a major factor in the isolation of fungal associates (e.g., whereas some media yield the highest number of isolates, other media recover the highest number of species [66, 67]). Therefore, it is of utmost importance to employ culture-independent techniques, such as next-generation internal transcribed spacer (ITS) amplicon sequencing, to further characterize the entire diversity of fungi associated with octocorals. The lack of comprehensive fungal reference databases, however, currently limits these efforts.

## Viruses

Research on coral-associated viruses is still in its early stages [68] and only two studies on the viromes of the *Gorgonia ventalina* have been published to date [69, 70]. The main viral groups found on gorgonians are phages of heterotrophic bacteria and cyanobacteria, but also double stranded DNA-viruses from the Phycodnaviridae family [69]. A more recent study found that this viral family was, however, not very abundant, in contrast to the phages, but also revealed members of the Parvoviridae, Totiviridae, and Circoviridae families [70]. While phages infect the bacteria and may be important regulators of the coral-associated bacterial communities, the role of the other viruses in the holobiont is unclear. Phycodnaviruses are known to infect eukaryotic algae and have been found in both healthy and bleached corals [71], as well as *Symbiodinium* cultures [71, 72]. However, their increased abundance in bleached corals [73] implicates them in the destruction of the algal symbiont *Symbiodinium*. Totiviridae use fungi (or protozoans) as their host and may be important in fungal diseases that have significantly affected *G. ventalina* populations, potentially by impacting the virulence of the fungal pathogen. Circoviridae are also commonly present in corals, particularly in diseased colonies [74], but their role is still unknown. Parvoviridae are known to infect numerous marine animals and infections can be asymptomatic, but also cause significant mortality. Their role in *G. ventalina*, however, remains to be elucidated. While two studies have identified some of the main viral groups within the octocoral virome, nothing is known about their role in holobiont functioning. As the role of viruses in coral holobiont health is becoming increasingly recognized, further studies on octocoral viromes and the role of viruses and phages in microbiome regulation and disease is warranted. One of the main challenges in virome research, however, is that the amount of viral nucleic acids present in samples is generally too low for sequencing library construction, requiring prior amplification of the viral genomic material. Although nucleic acid extraction protocols and whole genome amplification (WGA) may introduce biases (e.g., exclusion or overrepresentation of certain viral families), recent technological advancements have resulted in coral virome generation protocols that minimize such biases [75]. Optimizing and applying these protocols to octocorals will be the next phase, to better understand the role that viruses play in the holobionts of octocorals.

## Bacteria

Bacterial communities associated with octocorals have received significantly more attention than fungi and viruses, particularly in the Caribbean and the temperate waters around Europe, where octocoral populations have been significantly impacted by disease outbreaks (discussed in the next section). Generally, the bacterial richness and diversity in octocorals is lower when compared with those in scleractinian corals [76–78], which could make them more suitable model organisms for studying the function and evolution of coral–microbe symbioses. Most studies on tropical and deep-sea octocorals, however, have each focused on different hosts, and the most comprehensive datasets currently available focus on temperate gorgonians residing in the Mediterranean Sea. Therefore, we will summarize the findings from studies on these temperate gorgonians and discuss the commonalities and differences with their tropical and deep-sea relatives when information is available.

In the Mediterranean Sea, studies have focused on the iconic precious red coral *Corallium rubrum* (family Coralliidae (Lamouroux, 1812), sub-order Scleraxonia (Studer, 1887)) and the soft gorgonians from sub-order Holaxonia (Studer, 1887) belonging to the genera *Paramuricea* (family Plexauridae (Gray, 1859)), *Leptogorgia*, and *Eunicella* (family Gorgoniidae (Lamouroux, 1812)). The assessment of spatial and temporal differences in the gorgonian-associated bacterial communities has greatly facilitated our understanding on which bacteria compose the “core microbiome” and are likely essential to the holobiont. In-depth analyses of all Holaxonia species studied to date have revealed that their bacterial assemblages are highly dominated by Proteobacteria. For example, bacteria belonging to the Oceanospirillales genus *Endozoicomonas* can make up to over 96% of an octocoral’s bacterial assemblage [19, 20, 76, 78–82]. In addition, bacterial associates consistently found on various temperate Gorgoniidae are (in order of relative abundance) Cellvibrionales BD1-7 (previously Alteromonadales), *Mycoplasma*, *Aquimarina*, *Granulosicoccus*, and *Vibrio* species [19, 20, 76], while *Paramuricea clavata* was found to harbor a significant number of bacteria belonging to the candidate phylum NPL-UPA2 [19]. Interestingly, the bacterial assemblages of *C. rubrum* are quite unique within the phylum Cnidaria, being primarily composed of Spirochaetales, Oceanospirilalles family ME2 and Parcubacteria, and only a minor contribution of *Endozoicomonas* [20, 83]. While our knowledge on the composition of the octocoral microbiota has steadily increased, the exact role of these bacteria within the holobiont is currently unknown. Based on the functions of related bacteria and the recent whole genome sequencing of a few species, it has, however, been suggested that they are involved in (1) the acquisition and provision of nutrients, for example, through nitrogen fixation, carbon, nitrogen, and sulfur cycling, the synthesis of amino acids as well as aiding in food digestion, and (2) the regulation of the composition of the microbiota through the secretion of antibiotics and occupying functional niches to prevent the entry of pathogens. Below, we will describe the various bacterial taxa commonly found in the microbiota of healthy octocorals (Table 2).

**Table 2:** Overview of the bacteria most commonly found within octocoral holobionts. Taxonomy of the bacteria and which octocorals they associate with and their potential function are listed

Phylum	Class	Order	Family	Genus	Octocoral host	Potential function	Ref.
Actinobacteria	Actinobacteria	Propionibacteriales	Propionibacteriaceae	*Propionibacterium*	*Anthothela* spp. *Corallium rubrum* *Paramuricea* spp.	Unknown –Zooxanthellate corals: coral–*Symbiodinium* symbiosis	[20, 82, 93, 94, 121]
Bacteroidetes	Flavobacteriia	Flavobacteriales	Flavobacteriaceae	*Aquimarina*	*Antillogorgia elisabethae* *Eunicella spp.* *Leptogorgia sarmentosa*	Nutrient cycling Nitrogen cycling (denitrification) Carbon cycling (chitin degradation) Sulfur cycling (sulphate reduction) Microbiome regulation	[20, 89, 101]
Chlamydiae	Chlamydiae	Chlamydiales	Simkaniaceae	Candidatus *Fritschea*	*Eunicella* spp. *Leptogorgia sarmentosa*	Unknown	[20, 76]
Parcubacteria	–	–	–	–	*Corallium rubrum*	Unknown	[20, 83]
Proteobacteria	Alphaproteobacteria	Rickettsiales	Rickettsiales *incertae sedis*	Candidatus *Lariskella*	*Eunicella* spp. *Leptogorgia sarmentosa*	Unknown	[20, 76]
	Gammaproteobacteria	Cellvibrionales	Spongiibacteraceae	BD1-7 clade	*Corallium rubrum* *Eunicella* spp. *Leptogorgia sarmentosa*	Unknown	[20, 76]
		Oceanospirillales	Hahellaceae	*Endozoicomonas*	*Antillogorgia elisabethae* *Corallium rubrum* *Erythropodium caribaeorum* *Eunicella* spp. *Eunicea fusca* *Gorgonia ventalina* *Leptogorgia sarmentosa Lobophytum pauciflorum* *Paramuricea clavata* *Plexaura* sp. *Sarcophyton* sp. *Sinularia flexibilis*	Nutrient acquisition Nitrogen cycling Carbon cycling Sulfur cycling Amino acid synthesis Microbiome regulation	[19, 20, 76, 77, 83, 89–93, 257]
			ME2	–	*Corallium rubrum*	Unknown	[20, 83]
		Vibrionales	Vibrionaceae	*Vibrio*	*Corallium rubrum* *Eunicella* sp.	Putative pathogen (*V. shiloi*) Food digestion (*V. gigantis*-related)	[19, 20, 77, 105, 113–115]
		Chromatiales	Granulosicoccaceae	*Granulosicoccus*	*Eunicella* spp. *Leptogorgia sarmentosa*	Unknown	[20, 76]
Spirochaetae	Spirochaetes	Spirochaetales	Spirochaetaceae	*Borrelia*	*Corallium rubrum*	Unknown	[20, 83]
				*Spirochaeta*	*Anthothela* spp. *Corallium rubrum* *Lobophytum pauciflorum* *Muricea* spp.	Unknown	[20, 83, 91, 103, 121]
			Leptospiraceae	*Leptospira*	*Corallium rubrum*	Unknown	[20, 83]
Tenericutes	Mollicutes	Mycoplasmatales	Mycoplasmataceae	*Mycoplasma*	*Cryogorgia koolsae* *Eunicella* spp. *Leptogorgia sarmentosa* *Muricea* spp. *Pennatula phosphorea Plumarella superba* *Pseudoplexaura porosa* *Pteroeides spinosum*	Unknown Commensal feeding on captured prey	[19, 20, 102–106]
		Entomoplasmatales	Entomoplasmatales i*ncertae sedis*	Candidatus *Hepatoplasma*	*Eunicella* spp. *Leptogorgia sarmentosa* *Muricea* spp.	Unknown –Potentially copepod prey symbionts	[20, 103]

*Endozoicomonas* (order Oceanospirillales, family Hahellaceae) is commonly associated with a diverse range of marine organisms [84–88] and appears to be also one of the main constituents of the holobionts of gorgonians [19, 20, 77, 89, 90] and other octocorals [91–93] in the tropics, as well as in Antarctic waters, but is absent in some [94]. Because of its common association and the observed localization of these bacteria in aggregates within the tissues of corals [95] and possibly gorgonians [96], there appears to be an intimate biological integration between *Endozoicomonas* and corals. Many studies have tried to understand the role of *Endozoicomonas* in holobiont health (reviewed in [97]), providing indications that it may be involved in essential processes for holobiont functioning, such as nutrient acquisition (nitrogen and carbon recycling, methane and sulfur cycling, synthesis of amino acids) and bacterial community regulation via secondary metabolite production and competitive exclusion. In contrast to most other octocorals, the dominant Oceanospirillales members in the red coral *Corallium rubrum* microbiome belong to the family ME2 (up to 20%). Although their function is still unknown, their taxonomic relationship may indicate a similar role as *Endozoicomonas* [20, 83].

Cellvibrionales BD1-7 [98] are believed to be oligotrophs that may use light to generate ATP via proteorhodopsin proton pumps as an alternative energy source for mixotrophic growth [99, 100], but as they are the second most abundant bacterial taxon in Mediterranean gorgonians, they likely provide significant benefits to the holobiont. Another genus specialized for survival in oligotrophic conditions and commonly associated with a tropical [89] and various temperate gorgonians [20] is *Aquimarina*. Genome analysis of an *Aquimarina* symbiont isolated from *Eunicella labiata* [101] has revealed that it possesses a remarkable capacity to cycle nutrients: nitrogen (denitrification), sulfur (assimilatory sulphate reduction), and carbon (chitin degradation). In addition, it has a large arsenal of genes related to defense as well as for the production of antimicrobial compounds. Overall, this indicates that *Aquimarina* may play a role in nutrient acquisition and cycling, and microbiome structuring. However, the importance of these generally low abundant bacteria [20] for holobiont health remains to be investigated.

While *Mycoplasma* has generally been considered an intracellular parasite, it has been suggested that they are mutualists or commensals in temperate and deep-sea gorgonians [19, 20, 102–104] and sea pens [105], where they can be found in high abundance. *Mycoplasma* spp. may not be exclusive to soft corals from these environments, as they were recently also found in two tropical species [106]. In the cold-water scleractinian coral *Lophelia pertussa*, detailed studies on its *Mycoplasma* associates showed that they were in fact located extracellularly next to the spirocysts, suggesting that they opportunistically benefit from hemolymph leaking from prey captured by the animal, without affecting host health [107]. However, whether the octocoral-associated *Mycoplasma* have a similar role remains to be seen, as phylogenetic analysis showed that they form a different cluster from those associated with *L. pertusa* [102], and even closely related species may not perform the same functional roles. The origin and function of *Hepatoplasma*, a candidate genus within the Tenericutes, is also unclear. Although present in the microbiota of a number of temperate gorgonian species [20, 103], it may originate from planktonic arthropod prey as members of this genus are ectosymbionts of isopods [108, 109]. *Granulosicoccus* and members of the candidate genera *Lariskella* and *Fritschea* are also commonly found on temperate gorgonians [20, 76] and other cnidarians [110–112], but no functions have been identified yet.

One of the more striking findings across various studies has been the isolation and consistent presence of *Vibrio* bacteria in the microbiome of octocorals, including gorgonians [19, 20, 77, 113–115] and sea pens [105]. Although some *Vibrio* spp. are mutualistic, many have been implicated in disease, including outbreaks affecting gorgonians in the Mediterranean as well as tropical reef-building corals. Indeed, sequences matching the coral pathogen *V. shiloi* were present year-round in healthy specimens of various Mediterranean gorgonians [20], suggesting that it may be an opportunist rather than a specialized pathogen. However, not all gorgonian-associated *Vibrio* may be pathogens. For example, a *Vibrio* sp. that is a relatively low abundant but common (and in some cases core) member of the bacterial assemblages of nearly all investigated Mediterranean gorgonians and the red coral [20] is most closely related to *Vibrio gigantis*. This putative symbiont in Mediterranean clams [116] and sea cucumbers [117] likely aids its host in food digestion and belongs to the “Splendidus” clade that harbors both pathogenic and non-pathogenic *Vibrio* spp. Analysis of the genome of a *V. gigantis*-related bacterium (99.8% identity) isolated from *Eunicella verrucosa* suggested that it is indeed likely a generalist and opportunistic commensal symbiont [113].

Spirochaetes have received relatively little attention in the field of coral microbial ecology, probably due to their low abundance in tropical hard corals [17, 118, 119], cold-water corals [120], sea pens [105], and deep-sea soft corals [102, 104]. Recently, their potential relevance in coral holobiont health was, however, recognized when the bacterial communities of the red coral *Corallium rubrum* were found to be consistently composed of up to 70% Spirochaetales, taxonomically assigned to the genera *Spirochaeta*, *Borrelia*, and *Leptospira* [20, 83]. Since then, high abundance of *Spirochaeta* has been observed in the temperate gorgonian *Muricea californica* (up to 64%) [103], deep-sea *Anthothela* spp. [121], and the tropical soft coral *Lobophytum pauciflorum* (~ 43%) [91], while *Leptospira*-related sequences are commonly found in most Mediterranean gorgonians [20]. Despite their ubiquity and high abundance in at least a few soft coral species, the importance of these bacteria in holobiont functioning is still unknown. The order Spirochaetales contains many pathogens, but various species are known mutualists aiding in food digestion and fixation of nitrogen [122] and carbon [123] into bioavailable nutrients for the host. Even so, the role of Spirochaetales in octocoral holobionts remains unclear.

Another interesting feature of the microbiota of *C. rubrum*, is the presence of members of the phylum Parcubacteria. These bacteria represent up to 10% of the bacterial assemblages of the red coral [20, 83], but have thus far not been described as a symbiont of macro-organisms. In fact, members of this largely unknown phylum have been found primarily in anoxic conditions [124]. Genomic studies have indicated that Parcubacteria have a severely limited metabolic capacity [125] and likely rely for most of their nutrients on their host. Despite this reliance, their specialized lifestyle appears to be of a non-parasitic symbiotic nature, but the benefits to the holobiont are far from clear.

In contrast, Actinobacteria have been found in numerous studies on gorgonians, particularly from the deep sea [20, 82, 93, 94, 102, 121, 126, 127]. Recently, Actinobacteria from the *Propionibacterium* genus were implicated in the scleractinian coral-*Symbiodinium* symbiosis [22], but the presence of *Propionibacterium* in the absence of *Symbiodinium* in these gorgonians suggests their role may be different. Bacteroidetes, particularly Cytophaga and Flavobacteriia, [20, 91, 93, 104, 126, 128] may be important in the carbon cycling. These generally low abundant, but ubiquitous, gorgonian bacterial symbionts may aid in the degradation of complex organic molecules, such as the chitin from the exoskeleton of zooplankton [127].

While many studies have investigated the microbes living in association with octocorals and found numerous different taxa, our speculation on their function is based on their phylogeny and extrapolation of their role in the environment or other host organisms to the coral holobiont. While initially used, culture-based techniques have often provided significantly different assessments of bacterial community composition compared with culture-independent techniques, rarely picking up the dominant species and often overestimating the number of *Vibrio* spp. [77, 114, 115, 126]. However, recent culture and isolation efforts, and subsequent whole genome analysis of coral-associated bacteria has greatly facilitated our understanding of some potentially important octocoral symbionts, such as *Endozoicomonas euniceicola* and *E. gorgoniicola* [129], *Aquimarina* [101], *Pseudobacteriovorax antillogorgiicola* [130], and a *Vibrio* sp. [113]. Besides, certain characteristics are commonly assigned to certain taxa that may not always be true, for example, members of the genus *Vibrio* are often considered pathogens, but many *Vibrio* spp. may in fact be commensals or even mutualists. Resolving the function of the microbes within the coral holobiont should be our current priority, because only then will we truly increase our understanding on coral-microbe symbioses.

## Dominance in microbial assemblages

An interesting aspect in the octocoral microbiota is that it is often dominated by a few core microbiome operational taxonomic units (OTUs). This was observed initially in the temperate gorgonian *Paramuricea clavata*, whose bacterial communities were dominated (up to 91%) by one *Endozoicomonas* OTU [82], while other OTUs of this genus were present at low abundance. These results were confirmed later with similar observations made in the tropical octocorals *Lobophytum pauciflorum* [91] and *Erythopodium caribaeorum* [93] and several other Mediterranean gorgonians [19, 20]. In fact, this was not only true for *Endozoicomonas*, but also for NPL-UPA2 in *P. clavata*, the *Spirochaeta* in *L. pauciflorum*, and all main taxa in *Corallium rubrum* (Spirochaetales genera, Parcubacteria, and Oceanospirillales ME2) [20, 83]. Overall, this shows that octocoral hosts appear to have a preference for particular species from different taxa, but still harbor a large pool of very low abundant species (e.g., in Mediterranean gorgonians 669 of the 1512 OTUs were *Endozoicomonas*, but over 99% were very low abundant). These highly structured microbiota compositions suggest strong host-driven control of its microbial partners. While the relevance of maintaining such a high diversity of bacteria that are closely related to the main representative of a taxon at a low abundance is unclear, it may allow the host to change its main symbiont to a related species/strain that performs better under certain conditions to maintain holobiont physiological functions. There may be some indications of this principal of “symbiont shuffling” in the microbiota of *Eunicella verrucosa* and *Leptogorgia sarmentosa* at disturbed and undisturbed locations [20]. However, this has thus far only been shown to occur in hard corals, which changed their *Symbiodinium* endosymbionts in response to thermal stress to more heat-tolerant types [131].

## Spatial and temporal variability in associated microbiota

Currently, it is still difficult to assess the stability of the healthy octocoral microbiota and identify the most important core microbial symbionts, because most studies to date have focused on a limited number of species, sampled at one time point and/or from one geographical location. Spatial and temporal surveys of gorgonians from the Mediterranean have shown very little variation between relatively undisturbed locations and over time [19, 20, 81–83], even at the 97% OTU level. In fact, most of the variation observed could be attributed to changes in the abundance of core microbes [19, 20, 83]. In one study, however, *Endozoicomonas* transiently disappeared from the *Paramuricea clavata*-associated microbiota and was replaced by *Paenibacillus* and other bacteria from various taxa over a large geographical area at one time point [82]. It is unclear what may have caused this major shift as neither environmental anomalies nor any adverse health effects were observed. Interestingly, the microbial assemblage returned to an *Endozoicomonas-*dominated state again the next season, showing the selection for *Endozoicomonas* by the host. In contrast, significant changes in the microbiota of *Gorgonia ventalina* were observed during and following a thermal anomaly in 2010 [132]. Although the main taxa remained dominant, clear patterns were difficult to discern. Whether this shift adversely affected holobiont functioning or is a case of acclimation to thermal stress is unknown.

Studies on spatial microbiome variability are crucial to investigate the core microbiome. In addition, they have shown some interesting patterns in both gorgonians [19, 20, 83] as well as reef-building scleractinian corals [18], revealing that in addition to (1) the core microbiome, there are (2) locally stable microbial associates (LSMA; microbes consistently associated with a coral at a given location in addition to the core microbes) and (3) transient microbes. While the core microbiome is stable at all times, the composition of the LSMA consortium is different at each location, suggesting that adjustments in the octocoral microbiota could be a form of phenotypic plasticity that allows acclimation to local conditions. The relative stability of the bacterial communities in most octocorals suggests they are under strong host control, but the potential of microbiota plasticity in octocorals is unknown. In the Mediterranean, it appears that *L. sarmentosa* has a more flexible microbiome, while it may be more strictly defined in *Eunicella* species [19, 20]. To what extend microbiota plasticity allows a species to inhabit a larger range of environmental conditions remains to be investigated.

## Disturbances and acclimation

Analyses of the bacterial community have also revealed potential local impacts of anthropogenic origin (e.g., pollution, sedimentation, and mechanical damage) on gorgonian microbiome composition [19, 20, 78, 79]. In *P. clavata*, human impacts were found to result in a decrease in abundance of the main *Endozoicomonas* OTU, which may have opened up niches to be colonized by pathogenic *Vibrio* spp. [78]. Reduced *Endozoicomonas* abundances have generally been considered characteristic of stress in corals [112, 133–136]. Disturbances in the microbial assemblages of other Mediterranean species, showing a reduced contribution of the core microbiome but higher diversity including potential pathogens, were recently also attributed to polluted freshwater influxes from rivers and municipal sewage or submarine ground water discharges [19]. However, a more recent study nuanced these results finding that the local impacts are highly host species-dependent and raising questions about what may constitute a “healthy” microbiota [20]. Specifically, they found that the abundance of the LSMA and core microbes, in particular the most abundant *Endozoicomonas*, was significantly reduced in *E. verrucosa* at a site with high anthropogenic and terrestrial impacts compared to a relatively undisturbed site, but the exact opposite pattern was found in *L. sarmentosa* [20]. The fact that the abundance of this *Endozoicomonas* was differentially affected between two sympatric host species at the “disturbed” location shows that its viability or competitive potential was not affected by the environmental conditions, but was rather likely under host control. As such, care should be taken when linking the composition of coral-associated microbial assemblages to stress as differences may also represent acclimation and not all bacteria belonging to a taxa (e.g., *Vibrio*) harboring some pathogens are pathogenic.

Although changes in the octocoral microbiota have been linked to environmental and anthropogenic stressors, only one study has tried to establish causal links and came up short. They found that the bacterial communities of *Lobophytum pauciflorum* (Spirochaetes- and *Endozoicomonas*-dominated) were unaffected by temperature (31 °C) and acidification (pH 7.9) stress [91], confirming the relative stability of octocoral microbial assemblages. However, one point that should be considered in the design of experimental studies focused on linking microbiome function to particular stressors is that the microbiome in aquaria may be different than in the natural environment [91], an observation also made for scleractinian corals and other marine invertebrates. In contrast to scleractinian corals [137], physical injury did cause a change in the microbiota of the Caribbean gorgonians *Eunicea flexuosa* and *Pseudoplexaura porosa*. Notably, a decrease in *Endozoicomonas* near the lesion site was observed [106], which might be linked to a delay of colonization of the recovering tissues by bacteria from this genus.

## Co-diversification and inheritance

The consistent associations of specific OTUs with octocorals through space and time show the intricate relationships between these microbes and their hosts. Indeed, phylogenetic analyses at the OTU level across multiple gorgonian species from the Mediterranean Sea have shown that each host species harbors *Endozoicomonas* phylotypes belonging to different monophyletic clades [19, 20, 80, 81], but that species from the same family or genus share the same phylotypes in their microbiota [19, 20, 81]. The finding that the phylogenetic tree of the *Endozoicomonas* symbionts corresponds with the systematic classification of gorgonians suggests that co-diversification between these microbial symbionts and their hosts may have taken place [80, 81]. In fact, similar observations were made when the core microbiome as a whole was considered [19, 20]. These host-microbiota associations are therefore probably ancient and have been conserved through evolutionary times. However, these phylosymbiotic signals appear to end at the family/genus level, as there is significant overlap in OTUs between gorgonian species within the same family, although *Leptogorgia sarmentosa*’s microbiota showed some compositional difference with *Eunicella* species [20]. Interestingly, a recent study showed that there is in fact an incomplete phylogenetic separation of the *Eunicella* species and that there is potential for hybridization [138]. As such, the lack of divergence in the microbial assemblages between *Eunicella* spp. may be linked to the yet limited evolutionary divergence between these gorgonian species, and it would be interesting to observe how these associations will develop over time, what the hybrid holobiont composition is, and whether differences on the bacterial strain level may already exist. One discrepancy, however, is the difference in *Endozoicomonas* species found associated with *Gorgonia ventalina* and *Eunicella* spp., which both belong to the Gorgoniidae family [80]. However, these octocoral species are geographically separated by the Atlantic Ocean, while *L. sarmentosa* and *Eunicella* spp. are not, and this taxonomic family has been found to be polyphyletic [139–141], providing potential explanations for these observations.

The consistent, but host-specific, octocoral-microbe associations also raise questions concerning the mode of transmission. As most Mediterranean gorgonians are brooding (i.e., larval development occurs internally), vertical transmission of the bacterial symbionts to offspring is likely and has been shown to generate species-specific associations and co-diversification of the partners in a symbiosis [142]. Vertical transmission of symbionts is known to occur in the brooding scleractinian coral *Porites astreoides* [143], while horizontal transmission (i.e., uptake from the environment) is more likely to occur in broadcast spawning corals [144, 145]. Detailed studies into the transmission mode of microbial symbionts in octocorals would significantly increase our understanding on their inheritance and the evolution of coral symbioses.

## Octocoral diseases

**Fig. 3:**
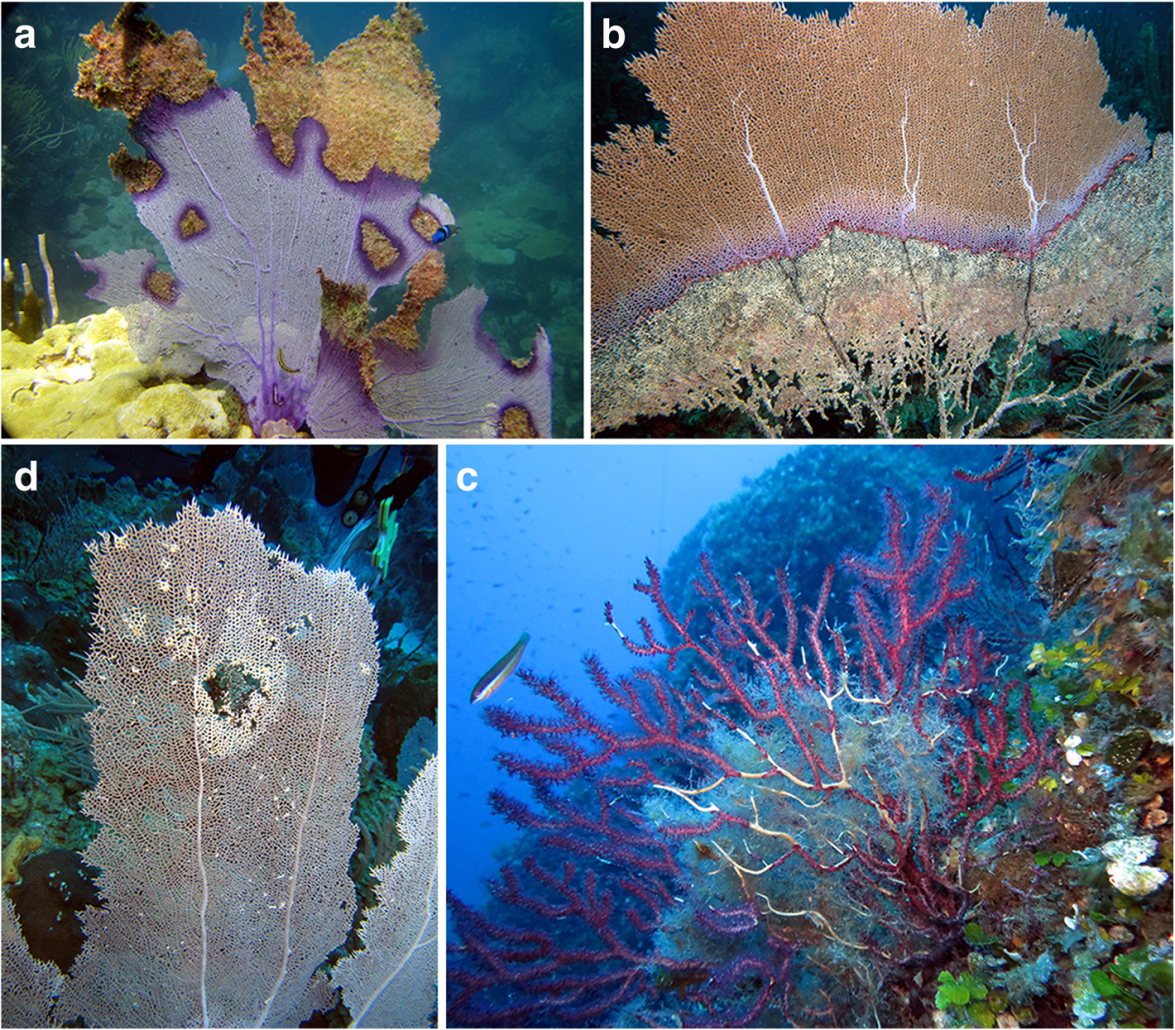
Octocoral diseases from the Caribbean and the Mediterranean Sea. **a** Aspergillosis, **b** Red Band Disease, **c** Octocoral *Vibrio* syndrome, and **d** *Gorgonia* Wasting Syndrome (photos **a**, **b**, and **d** by Ernesto Weil and photo **c** by Carlo Cerrano)

Disease outbreaks have affected many marine organisms worldwide in recent decades [146–148] and can produce major changes to ecosystem composition, structure, and function as observed on coral reefs [149–151]. The main drivers of the increased incidence, prevalence, virulence, and emergence of new marine diseases are likely related to changes in the environment linked to climate change [152]; however, local anthropogenic factors are known to exacerbate the effects [60, 153–155]. Disease is an interaction between a host organism, pathogen, and the environment. Changing environmental conditions, such as higher than normal seawater temperatures, may compromise the host (physiology and immunity), making it more susceptible to pathogens and causing shifts in the associated microbiota [156]. Currently, 19 different diseases have been identified that are known to affect octocoral populations worldwide. Octocoral diseases were recently expertly reviewed in detail by Weil et al. [56], and we will therefore only provide a brief overview of this subject focused on the main microbial diseases (Fig. 3; Table 3). A commonality among all octocoral diseases is that their prevalence seems to increase with higher seawater temperatures.

**Table 3:** Overview of the main octocoral diseases, the putative pathogens and the octocoral genera/species affected. If multiple octocoral species from the same genus were affected, only the genus name is provided

	Region	Pathogen	Octocoral affected
Microbial diseases			
Aspergillosis	Caribbean	*Aspergillus sydowii* *Aspergillus* spp.	*Gorgonia* spp. *Pseudopterogorgia* spp. *Plexaura* spp. *Pseudoplexaura porosa* *Plexaurella* spp.
	Pacific		*Pacifigorgia* spp.
Red Band Disease	Caribbean	Polymicrobial mat – primarily cyanobacteria *Ocillatoria* sp.	*Gorgonia* spp. *Plexaurella nutans*
Black Band Disease	Caribbean	Polymicrobial mat – cyanobacteria *Phormidium corallyticum,* sulphate-reducing *Desulfovibrio*, sulfide-oxidizing *Beggiatoa*	*Gorgonia* spp. *Pseudopterogorgia* spp. *Plexaurella* spp. *Erythropodium asbestinum*
Octocoral *Vibrio* Syndrome (OVS)	Mediterranean	*Vibrio coralliilyticus*	*Eunicella* spp. *Paramuricea clavata*
Fungal-Protozoan Syndrome *Possibly secondary infection following OVS	Mediterranean	Fungus genera *Trichoderma*, *Clodosporium*, *Penicillium* Unidentified protozoa	*Eunicella* spp. *Leptogorgia sarmentosa* *Paramuricea clavata* *Corallium rubrum*
Black Necrotic Syndrome	Pacific	Possibly *Penicullium* fungus	*Isis hippuris*
Multifocal Purple Spots (previously labyrinthulomycosis)	Caribbean	*Aplanochytrium* protozoan *Sphaerippe* copepod	*Gorgonia* spp.
Wasting Syndromes (WS)	Caribbean	Unknown pathogens	
Gorgonian WS			*Gorgonia* spp. Plexaura nutans *Erythropodium asbestinum*
*Briareum* WS			*Briareum* spp.
*Phyllogorgia* WS			*Phyllogorgia dilatata*
*Erythropodium* WS			*Erythropodium asbestinum*
Bleaching Necrosis (BN)	Caribbean	Unknown pathogens	
*Briareum* BN			*Briareum* spp.
*Erythropodium* BN			*Erythropodium caribaeorum*
Other diseases			
Growth anomalies Hyperplasia, hypoplasia No documented mortality	Caribbean	Endolithic algae – *Entocladia endozoica*	*Gorgonia* spp. *Pseudopterogorgia* spp. *Plexaura* spp. *Pseudoplexaura* spp. *Plexaurella anceps*
Bleaching Loss of zooxanthellae	Caribbean	High seawater temperatures	*Gorgonia* spp. *Pseudopterogorgia* spp. *Plexaura* spp. *Pseudoplexaura* spp. *Plexaurella* spp. *Briareum* spp. *Muricea* spp. *Eunicea* spp. *Erythropodium asbestinum* *Pterogorgia citrina* *Muriceopsis flavida*
	Pacific		*Isis hippuris* *Lobophyton* spp. *Sarcophyton* spp. *Sinularia* spp.

The octocoral disease aspergillosis (Fig. 3a) has been the most devastating in the Caribbean and Eastern Pacific [60, 157, 158] and is currently considered a chronic disease in the wider Caribbean region [62]. Aspergillosis disease dynamics vary across locations and reefs, indicating that local biotic and abiotic factors influence disease prevalence [60, 61]. Differential disease susceptibility (e.g., host resistance) among octocoral species has also resulted in changes in octocoral communities [159, 160]. The fungus *Aspergillus sydowii* was identified as the pathogen, but various other *Aspergillus* species have been shown to cause similar disease signs [64]. Infiltration of the fungal hyphae into the coenenchyme tissue results in degradation of the tissue exposing the skeleton, which is then rapidly colonized by other micro- and macro-organisms [60]. Characteristic for aspergillosis is the purpling of the tissue surrounding the lesions due to activation of the melanisation cascade, a component of the immune response (discussed in the next section) exhibited by the coral to prevent further progression of the fungal infection. Contrastingly, growth anomalies rarely cause mortality in octocorals [56]. Although detected throughout the Caribbean, the cause of these abnormal tissue structures (e.g., tumors, hyper- or hypoplasia) is unclear, but may be part of a general response against parasites, fungal, or algal infiltrations, competition, and damage/injury [161–164].

Black Band Disease (BBD) and Red Band Disease (RBD) (Fig. 3b) are two diseases that affect both hard [165] and soft corals [166–168] and seem to be temperature driven as they are more prevalent in warm summer months. The characteristic “band” is a polymicrobial mat that in scleractinian corals consists primarily of cyanobacteria [169, 170], combined with sulfur-reducing and sulfide-oxidizing bacteria in the BBD consortium [169]. The composition of the bacterial mats in the octocoral BBD and RBD has yet to be identified.

In the Mediterranean, two mass mortality events related to high seawater temperatures took place over large geographical areas in 1999 and 2003, affecting 60–100% of the gorgonian populations as well as many other benthic marine invertebrates [171–174]. The bacterium *Vibrio coralliilyticus*, which is known to cause disease in scleractinian corals in the Indo-Pacific, was identified as the putative pathogen in the 2003 outbreak, and the disease has been termed Octocoral *Vibrio* Syndrome (Fig. 3c) [172]. The disease manifests itself by mucus production by the gorgonian, followed by a loss of pigmentation and subsequently the coenenchyme tissue. Despite the lack of conclusive evidence, it is believed that this bacterium was also responsible for the mortality of octocorals in 1999. The fungal hyphae and protozoan ciliates found on diseased gorgonians [171] (responsible for the name Fungal-Protozoan Syndrome [175]) were likely secondary opportunistic parasites. *Eunicella verrucosa* colonies were also impacted by a disease with similar signs in southwest England between 2002 and 2006 [176].

Black Necrotic Syndrome is affecting gorgonians in the Pacific and is characterized by black necrotic areas along the branches, followed by rapid tissue and skeleton loss, leading to the fragmentation of the entire colony [177]. Although *Penicillium* fungi were isolated from lesions that contained high numbers of hyphae, it could not be proven that these microbes were indeed the disease-causing pathogens [177].

The effect of two parasitic diseases affecting the gorgonian *G. ventalina* classified as Multifocal Purple Spots (MFPS) on the physiological functioning of the coral holobiont is unknown, but the indication of an active immune response based on purpling of the tissues suggests the infection is indeed harmful. MFPS can be caused by ovoid protozoans form the genus *Aplanochytrium* [178, 179] and appears as small purple galls with the protozoans located inside. Larger MFPS galls containing one or two *Sphaerippe* copepods have recently also been described [180]. Parasitism of other octocorals (sea pens, deep-sea gorgonians) by endoparasitic copepods is, however, quite common [181, 182], but to what extend it affects host survival remains unclear.

Wasting Syndrome is another class of disease that has severely impacted various gorgonians, including species belonging to the genera *Phyllogorgia* [183], *Erythropodium* [56], *Gorgonia* (Fig. 3d) [184], and *Briareum* [55, 185, 186]. The disease is characterized by discoloration and disorganization of the tissues, ultimately resulting in decomposition with necrotic appearance. However, no putative pathogens have thus far been identified. In addition to these characterized diseases, other disease-resembling conditions have been observed on octocoral colonies. As little is known about the etiology and pathogens of octocoral diseases, there is an urgent need to rapidly investigate the disease-causing microbial consortia, isolate the suspected pathogens to fulfill Koch’s postulates, and develop diagnostic and management tools for the protection of the ecologically important octocoral assemblages.

## Octocoral immune responses

**Fig. 4:**
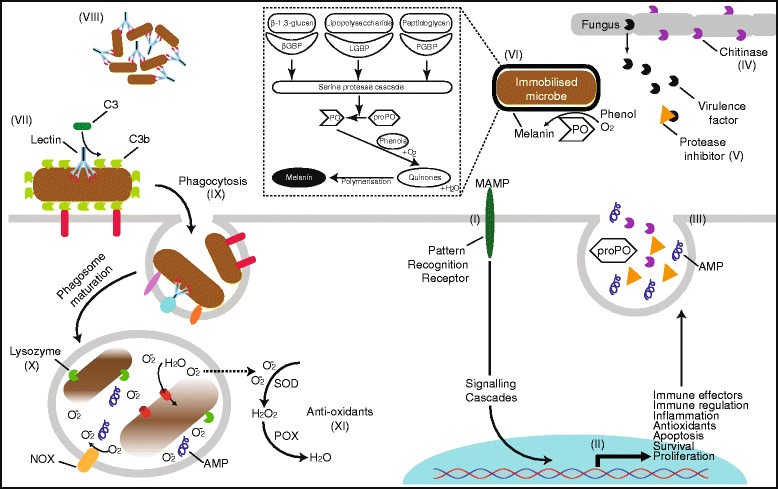
Current knowledge on the immune system of octocorals. (I) Microbe-associated molecular patterns (MAMP) are recognized by pattern recognition receptors (PRR), which subsequently activate signaling cascades that induce (II) expression of genes involved in the immune system. (III) Immune effector molecules are produced and secreted, including antimicrobial peptides (AMP). (IV) Chitinases degrade chitin, an important component of the cell wall of fungi. (V) The host also uses protease inhibitors to neutralize protease virulence factors secreted by pathogenic microbes. (VI) One of the main immune system components is the prophenoloxidase (proPO)-activating pathway. It is activated following the binding of MAMPs to their respective binding proteins (BP), leading to the activation of a protease cascade that ultimately cleaves proPO into PO. Subsequently, PO oxidizes phenolic compounds (e.g., dihydroxidephenylalanine) that undergo further non-enzymatic reactions to form a microbe-immobilizing barrier of melanin. Cytotoxic molecules are also formed during this process. Octocorals are also known to possess lectins, which can be used in (VII) the lectin-complement system that leads to the deposition of complement C3 on the target microbe, and/or to (VIII) aggregate microbes into large aggregates. Both systems facilitate (IX) the rapid phagocytosis of microbes following binding to lectin, C3-receptors or various scavenger PRRs. Once internalized, the phagosome matures and becomes microbicidal with (X) bacterial cell wall degrading lysozyme as well as AMPs and oxidative burst of reactive oxygen species (ROS). (XI) The ROS may be also damaging to the host cell and antioxidant enzymes, such as superoxide dismutase (SOD) and peroxidase (POX), are used to neutralize it

Although diseases have had a major impact on octocoral populations, these organisms have a remarkable immune defense capacity (Fig. 4). The first evidence of self and non-self recognition in cnidarians was presented in 1893 by Metchnikoff, the pioneer of cellular immunology, following his observation of an accumulation of amoebocytes around a splinter inserted in a scyphozoan [187]. This finding gave rise to extensive graft rejection studies using the gorgonian *Swiftia exserta* as a model. While autografts readily fused with the colony, allografts were rejected exhibiting tissue necrosis at the graft site and thereby showing the principle of histocompatibility in octocorals [188]. This response was likely mediated by the “granular amoebocytes,” which infiltrated the graft rejection areas and are also known to accumulate in tissue wounds in soft corals [189]. In addition, some populations of these cells have been shown to possess phagocytic capabilities [190] and are thus considered putative immune cells, similar to macrophages and neutrophils in vertebrates. Recent biochemical characterizations support this notion and indicate that *S. exserta* possesses at least three distinct immune cell types [191]. The authors hypothesize that invading microorganisms first encounter the immediate-responders, consisting of (1) “initial-encounter cells” in the ectoderm epithelium, which fight off the microbes with chemical oxidation and (2) “focal response cells” located directly under the epithelium, which are equipped with acid phosphatase and esterases. In addition, there is a population of secondary responders, called (3) *DOPA-oxidase-containing cells*, that migrate within the mesoglea towards lesions and also possess the potent microbicidal enzyme phenoloxidase and peroxidase. These secondary response immunocytes are likely the large population of specialized phagocytic immune cells observed infiltrating lesions 24 h post-injury, while the immediate responders arrive on the scene within 2 h [190].

These “granular amoebocytes” were, however, only truly known to be implicated in the antimicrobial immune response of octocorals following studies into the major aspergillosis outbreaks on *G. ventalina*. Dense aggregations of amoebocytes were observed in tissues infected by *Aspergillus sydowii*, with concurrent increased phenoloxidase activity and melanin deposition [192]. The melanin was found to form a thick band around the lesion sites and surround the fungal hyphae [193] to form a protective barrier and prevent further tissue infiltration by the fungus. Surprisingly, however, the amoebocytes did not appear to migrate towards the lesion in this gorgonian, but likely originated from stem cell-like undifferentiated amoebocytes that underwent significant proliferation near the site of infection [192].

Other inducible enzymatic defense mechanisms have also been described in octocorals. For example, the activities of two classes of antioxidant enzymes have been implicated in the coral immunity: peroxidase (POX) and superoxide dismutase (SOD). While SOD scavenges superoxide radicals (O_2_^−^) and converts it to O_2_ or hydrogen peroxide (H_2_O_2_) depending on the SOD enzyme type, POX neutralizes H_2_O_2_. Regulation of the oxidative stress during the antimicrobial oxidative burst is crucial to prevent tissue damage and can be used as a proxy to assess the organism’s immune system’s activity. Peroxidases are present in the secondary response cells in *S. exserta* [191], and their activity has been shown to be induced in response to injury and heat stress [194] as well as *A. sydowii* infections [195]. Both SOD and POX activity have been related to potent antifungal activity [195, 196]. Particularly relevant in antifungal defense in gorgonians may also be the destruction of the fungal cell wall through chitin degradation by chitinases [197], while the digestion of peptidoglycan by lysozyme-like enzymes may be important to fight off bacteria [196]. While attacking structural components of pathogens is an efficient defense mechanism, pathogens use virulence factors, such as proteases, to damage and subsequently infiltrate the host tissues. Inhibition of virulence factors may be another defense strategy employed by octocorals as protease inhibitors that inhibit the activity of such fungal protease virulence factors were recently described [198].

The majority of immune system studies in octocorals have focused on enzymatic defense systems. However, the availability of genomic and transcriptomic approaches have provided additional insights into the octocoral immune repertoire. Characterization of a lectin [199] and a C3-like molecule [200] suggests that the lectin-complement system, which facilitates efficient phagocytosis of microbes, may also be present in these organisms. Challenges of *G. ventalina* with an *Aplanochytrium* parasite further revealed that this gorgonian upregulates the expression of various pattern recognition receptors, whose signaling may be responsible for the increased levels of antimicrobial peptides (AMP) [178] that may play a role in the regulation of the associated microbial communities as has been demonstrated in *Hydra* [201].

The large inducible immune repertoire of octocorals suggests that these organisms possess significant capabilities to fight off infections. Nonetheless, as diseases have particularly impacted octocoral populations worldwide in recent decades, differences in environmental conditions and locations may also affect the immunocompetence of octocorals [196]. Although little is known about the relationship between the environment and the octocoral immune system, current studies have only detected increases in immune parameters under potential stress conditions. For example, increased levels of dissolved inorganic nitrogen positively correlated with chitinase and lysozyme-like enzyme activities [196], while increases in amoebocytes [192], protease inhibitor [198], and antifungal [202] activity have been observed under elevated seawater temperatures. However, under these conditions, microbial growth may be stimulated [202], requiring the host to invest in immunity. Prolonged microbial stress and resource allocation towards immunity may significantly reduce the energetic resources available and ultimately lead to an (immuno)compromised health state and disease. A link between reduced investment in the immune system and higher disease prevalence has been demonstrated, even within colonies. In gorgonians, growing tissues possess significantly higher levels of immunity compared with older tissues [196, 203], and disease modeling studies have demonstrated that this spatial within-host difference may explain the higher prevalence of disease in larger colonies and found that new infections are more likely to occur when hosts direct their immune responses to lesions at the expense of other healthy parts in the colony [204].

## Microbiome regulation

While most inducible cellular immune mechanisms found to date are likely used by the host in response to microbial infections, constitutive expression of compounds with antimicrobial properties may be used by the host organism to regulate its microbiome and keep pathogens out. Gorgonian tissue extracts have been tested extensively for antibacterial [114, 196, 205–207] and antifungal activities [196, 202, 203, 208, 209]. However, it is unclear which compounds are responsible for the bioactivity observed in these studies and which member within the holobiont produces these compounds. One of the main microbiome regulatory mechanisms in octocorals may be the host’s immune system (see the previous section). For example, the expression of antimicrobial molecules is modulated via pattern recognition receptors that monitor the microbiome and thereby regulate the composition of the associated microbiota, as has been demonstrated in other cnidarians [111, 201, 210].

**Fig. 5:**
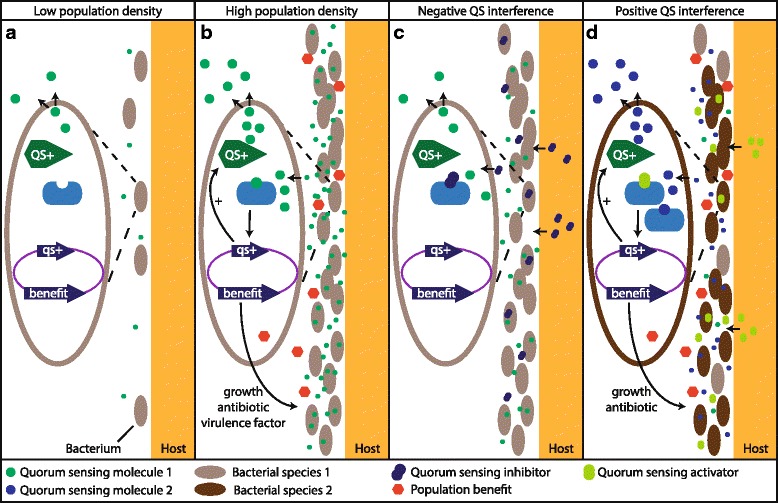
Quorum sensing and interference. **a** Bacteria produce quorum-sensing stimulating compounds (QS+), but due to low bacterial density and environmental conditions (e.g., diffusion, advection, degradation) the concentration does not reach levels sufficient to bind to the receptor. **b** At high population densities, the QS compounds reach sufficient levels to bind to its receptor leading to gene transcription and subsequently (1) increased production of QS+ signaling molecules and (2) population beneficial processes, such as cooperative growth and migration, secretion of antibiotics to reach effective concentrations for competition or in case of pathogens the production of virulence factors. **c**–**d** Host organisms have the capacity to interfere with QS, possibly species specific. **c** By secreting QS inhibiting compounds, bacterial population benefits can be counteracted, thereby reducing bacterial growth and potentially inhibiting pathogen virulence. **d** Host-induced QS activation in specific bacterial species may provide a growth advantage, selecting for those species. A balance of negative and positive QS interference may allow the host to regulate its associated microbiota. Other than intended bacterial species may, however, cheat and benefit from QS by other species without investing in QS themselves

Another strategy for microbiome regulation is interference with quorum-sensing (QS). QS is a microbial communication process using signaling molecules to mediate cooperative behaviors between related microbes (Fig. 5; expertly reviewed by Asfahl et al. [211]). However, a host organism may regulate its microbiome through QS interference, thereby stimulating or inhibiting the growth and functions of beneficial and potential damaging microorganisms, respectively (Fig. 5). A recent study in *Hydra* showed the importance of QS interference for microbiome regulation in cnidarians [212]. Through an enzymatic modification of *N*-acylhomoserine lactone (AHL) QS molecules, the host was found to manipulate bacterial QS, thereby changing gene expression patterns and inducing a phenotypic switch in the bacteria, which ultimately lead to reduced colonization of the host by specific bacterial symbionts. Octocoral extracts also contain compounds with QS regulatory properties [213–215], particularly diterpenes [213]. Several cembranoid diterpenes have been isolated from soft corals and were implicated in the inhibition of *N*-acylhomoserine lactone (AHL)-mediated QS, the best studied QS system in gram-negative bacteria, resulting in reduced biofouling [213, 216–218]. Interestingly, however, other cembranoid diterpenes and furanoditerpenes appeared to be QS mimics and possessed stimulatory properties [213]. While diterpenes contain lactone-rings used to bind the AHL receptors, it was demonstrated recently that a sterol abundantly present in the octocoral *Nephthea chabroli* may also efficiently stimulate AHL-type QS [213]. Using a range of QS inhibitory and stimulatory compounds specific for various microbes may allow octocorals to tightly regulate the composition of their microbiota and could explain the observed relative stability of soft coral-associated bacterial communities. However, some bacteria associated with octocorals, such as *Endozoicomonas*, have also been found to exhibit potent QS activity [213], providing another potential explanation why these bacteria are highly dominant in some gorgonians. In contrast, *Vibrio* species generally inhibited QS in biosensor species [213]. This potentially disruptive effect on QS in other members of the microbiota may be a competitive strategy of these potential pathogens to establish themselves in the microbiota of the host. As most QS interfering molecules have been characterized as part of natural product discovery efforts focused on finding antimicrobial compounds, QS stimulatory molecules may have been largely overlooked. More research into these compounds, their ecological role, and the interplay between inhibitory and stimulatory QS signaling will be required to fully understand the importance of QS interference by soft corals in their microbiome regulation.

In addition to QS interference, numerous secondary metabolites with antimicrobial, antiviral and antifouling activity have been isolated from soft corals (overview provided in [219]). Although these compounds are likely used by the host to eliminate unwanted microbes, prevent overgrowth by other benthic organisms, and maintain colony health, their ecological relevance for holobiont functioning remains to be elucidated. Interestingly, however, microbes are increasingly recognized to be involved in regulation of holobiont composition and defense as well. Various compounds have recently been extracted from microorganisms that were isolated from octocorals. For example, several phenyl ethers, anthraquinones and alkaloids with anti-fouling properties [220], and merosesquitepenoids, macrolides and alternariol derivatives with antibacterial [221, 222] and potentially antiviral activities [222], were derived from symbiotic fungi associated with soft corals. Various bacteria belonging to the Firmicutes, Actinobacteria, and Gammaproteobacteria isolated from tropical and temperate octocorals have also been shown to possess potent antibacterial and antifungal activities [223–225]; however, only the antimicrobial cyclic tetrapeptide Cereustatin A and two esters of *p*-hydroxybenzoic acid have so far been extracted and characterized [226]. Interestingly, the algal symbiont *Symbiodinium* was found to be a rich source of diterpenes in the gorgonian *Antillogorgia elisabethae* and *A. bipinnata* (previously belonging to the genus *Pseudopterogorgia* [227]), called pseudopterosins [228, 229]. While those molecules possess anti-inflammatory and antimicrobial activities, one study has implicated them in the regulation of a damage-inducible oxidative burst in cultured algal cells [230].

Taken together, soft corals and their microbial associates possess a range of molecules that affect the growth and survival of microorganisms. As such, the role of the microbial community in holobiont physiology and immunity should also be considered. The composition of a healthy symbiotic microbiome may be largely regulated via positive or negative quorum sensing interference, while antimicrobial compounds may be used to prevent pathogen infiltration, supporting the “coral probiotic hypothesis” [231]. As the majority of studies has been conducted on isolated microbes or extracted compounds, their ecological importance in coral microbiome regulation remains an avenue of research.

## Natural product discovery and challenges

Since the 1950s, when research into marine natural products started, sponges have been considered to have the highest potential for drug discovery. However, new technologies and increased efforts identified soft corals as a rich source of potentially bioactive secondary metabolites. Despite an initial surge in the testing and characterization of compounds extracted from octocorals [219], relatively few new compounds have been described in recent years [232]. Overall, more than 3500 bioactive molecules from octocorals have now been described and tested, with some promising drug leads (Table 4; reviewed in [219]). The vast majority of such compounds belong to the highly diverse classes of terpenes and terpenoids, particularly diterpenoids and cembranoids, as well as steroids and prostanoids. For example, *Plexaura homomalla* contains very high levels of prostaglandin A_2_, which has a predator-deterring effect [233]. Testing for medically relevant bioactivity revealed that many of those compounds possess anticancer, anti-inflammatory, or antimicrobial (e.g., antiviral, anti-ulcer, anti-malaria, anti-tuberculosis, or more general antibacterial and/or antifungal) properties. Especially from a pharmaceutical perspective, it is interesting to note that non-marine bacterial pathogens were generally more sensitive to gorgonian tissue extracts than marine bacteria [206, 214]. However, another consideration that should be further investigated are the ecologically and pharmaceutically relevant doses, particularly as some compounds with antibacterial activity have been shown to induce QS at low concentrations [213]. Although octocoral-derived compounds are not being used in the clinic yet, pseudopterosins derived from *A. elisabethae* are the main components of some cosmetic skincare products, such as Resilience by Estée Lauder, because of potential anti-aging effects due to their anti-inflammatory properties [234]. Other compounds have been found to have potent anti-fouling activity (Table 4), making them potentially suitable natural alternatives to tributyltin for fouling prevention on ships.

**Table 4:** List of 25 promising natural product compounds isolated from octocorals, showing the diversity of chemical compounds with various pharmaceutically relevant activities produced by a range of different octocoral species

Activity	Compound	Chemical	Origin	Region	Ref
Anti-inflammatory	Austrasulfone	Sulfone	*Cladiella australis*	Taiwan	[258]
	Simplexin E	Diterpenoid	*Klyxum simplex*	Taiwan	[259]
	Crassumolides A and C	Terpenoid	*Lobophytum crassum*	Taiwan	[260]
	Ergostanoids 1 and 3	Ergostanoid	*Nephthea erecta*	Taiwan	[261]
	Paralemnolin Q and S	Sesquiterpenoid	*Paralemnalia thyrsoides*	Taiwan	[262]
	Lobocrassin B	Cembranoid	*Lobophytum crassum*	Taiwan	[263]
Antitumor	Polyoxygenated gorgosterol (2–4)	Steroid	*Isis hippuris*	Japan	[264]
	Clavulones	Prostanoid	*Clavularia viridis*	Taiwan	[265]
	Bis(pseudopterane) amine	Dialkylamine	*Antillogorgia acerosa*	Bahamas	[266]
	Klysimplexin B and H	Diterpenoid	*Klyxum simplex*	Taiwan	[267]
	13-acetoxysarcophytoxide	Cembranoid	*Lobophytum crassum*	Taiwan	[268]
	Capilloquinol	Farnesyl quinoid	*Sinularia capillosa*	Taiwan	[269]
Antimicrobial	Curcuphenol	Terpenoid	*Antillogorgia rigida*	USA	[270]
	Lipids	Polyketide	*Sinularia* sp.	Russia	[271]
	Pseudopterosin X	Diterpenoid	*Antillogorgia elisabethae*	USA	[272]
Antiviral	Durumolide Q	Cembranoid	*Lobophytum durum*	Taiwan	[273]
	Lobohedleolide	Diterpenoid	*Lobophytum* sp.	Philippines	[274]
Antituberculosis	Bipinnapterolide	Terpenoid	*Antillogorgia bipinnata*	USA	[275]
	Homopseudopteroxazole	Diterpenoid	*Antillogorgia elisabethae*	USA	[276]
Antimalaria	Aberrarone	Diterpenoid	*Antillogorgia elisabethae*	Colombia	[277]
	Dolabellane	Diterpenoid	*Eunicea* sp.	Colombia	[278]
Anti-fouling	Homarine	Pyridine	*Leptogorgia setacea*	USA	[279]
	11-episinulariolide	Diterpenoid	*Sinularia flexibilis*	Australia	[280]
	Isogosterones A–D	Steroid	*Dendronephthya* sp.	Japan	[281]
	3β-methoxyguaian-10(14)-en-2β-ol	Sesquiterpenoid	*Echinogorgia pseudosassapo*	Taiwan	[282]

As secondary metabolites are generally present in the holobiont at relatively low concentrations, large quantities of organisms will be required. Such unsustainable harvesting is unwanted because of the potentially severe impacts on ecosystems. Due to the complex structures of soft coral-derived natural products, it is very difficult to produce these molecules synthetically in the laboratory. While aquaculture has been suggested as a viable alternative to natural harvesting as it has limited environmental impact, it is a relatively slow and intensive process. Culturing the microbes that produce natural product (NPs) of interest has also proven challenging as the production of the secondary metabolites depends significantly on the culture conditions and many of these microbes are yet uncultivable under laboratory conditions. Given these challenges in supply, the identification of the exact metabolic pathways and cloning of the genes involved into resistant prokaryotic or eukaryotic expression vectors for constitutive production would be ideal.

Overall, soft corals and their microbial associates are recognized as excellent sources for potentially interesting drugs and anti-fouling compounds. While small quantities are sufficient for initial screens and pre-clinical studies, major challenges in the supply of these molecules still need to be overcome, before they become feasible drug candidates for clinical applications.

## Future directions

Although octocorals function as ecosystem engineers in a wide variety of environments, they have received significantly less attention than scleractinian corals, whose physiology and holobiont composition have been extensively studied. Octocorals are, however, severely affected by pollution, disease, and global climate change threats, such as rising seawater temperatures [53], and therefore deserve further research, particularly at the holobiont level. Microbes are emerging as very diverse and flexible symbionts of corals and microbial processes are important for coral health and resilience to stress, but the functional role of these microbes within the coral holobiont is poorly understood. There are thus many questions at the forefront of discovery, some of those being the same as for scleractinian corals.

Concerning the octocoral-dinoflagellate symbiosis, one fundamental but still unknown aspect in this relationship is the importance of autotrophy versus heterotrophy in the energetic budget of octocoral species at the different seasons and under different environmental conditions. Such knowledge will be essential to understand how octocorals acquire energy to face stressful conditions occurring at the global and local scales. The contribution of *Symbiodinium* to the reproductive effort of octocorals under different stress scenarios will also be crucial for understanding the potential of these species to spread and colonize new environments. The increased frequency in bleaching events and seawater eutrophication cause coral mortality in tropical reefs worldwide [235]. Octocorals appear to have a lower bleaching susceptibility as well as a higher resilience to eutrophication compared to scleractinians, and phase shifts towards soft coral dominance has already occurred in some regions (Table 1). This higher resistance of octocorals compared to other coral groups has been attributed to their lower dependency on the autotrophic input of the dinoflagellate symbionts, replaced by a higher degree of heterotrophy [7]. However, not all octocorals can afford a reduction in autotrophic input [50]. More studies are thus necessary to unravel the energetic needs of octocorals and to estimate the cost of this symbiosis for mixotrophic species. For example, the stability of the symbiosis suggests that octocorals are more resilient to global warming than predicted, or on the contrary, that the host is unable to switch its symbionts towards more resistant ones [131]. To answer this question, there is a need to increase our knowledge on the resilience and recovery of octocoral species following bleaching events.

The microbial diversity associated with octocorals needs to be better characterized to identify those microbes that are essential to holobiont health and those that may impair holobiont functioning and cause disease. Knowing which microbes are (opportunistic) pathogens could be used to develop diagnostic tools to monitor soft coral populations and inform management strategies when changes in the octocoral microbiota towards a pathogenic state occur. Many soft corals harbor a microbiota of lower diversity and present a more defined and stable core microbiome than their scleractinian relatives [19, 20, 83, 91]. Such stable associations are particularly useful to study the functional role of the associated bacteria and show that octocorals may be a good model organism to study coral-microbe interactions. For example, *Endozoicomonas* symbionts are dominant in some octocoral species and the fact that multiple genotypes can be present in a single host suggests that the host may be able to alter its *Endozoicomonas* population to the environmental conditions. However, environmental stress tends to correlate with a decreased *Endozoicomonas* abundance, indicating that this bacterium likely plays a role in host fitness [19, 110, 134, 135]. The exact functions of this bacterial genus in coral holobiont symbiosis remain to be identified though. While *Endozoicomonas* has received significant attention due to its wide global distribution and associations with many marine invertebrates, the apparent equally important role of Spirochaetes and *Mycoplasma* in some octocorals, for example, should also be given substantial consideration. Metagenomic and metatranscriptomic approaches will allow us to reconstruct the genomes of those difficult-to-culture symbionts and assess the impacts of stressors on their functioning. However, technical challenges regarding the low recovery of microbial reads due to host contamination need to be resolved to allow this technique to be used cost-effectively. Another avenue of research that is vastly underexplored is the role of fungi, archaea, and viruses in octocorals. Research on these taxa in scleractinian corals is only in its early stages and, as a potential model, the associations between octocorals and those microbial taxa may provide important insights applicable to reef coral biology.

Progress can also be made on experimental and technological fronts. As the field of octocoral microbiome research is still in its infancy, we have the opportunity to benefit from the knowledge gained from other fields. For example, each 16S rRNA gene-targeting primer set is known to have an inherent bias towards certain taxa. We also observed significant differences in bacterial community composition associated with octocorals when using primers targeting the V5–V6 regions of the 16S rRNA gene or the V1–V2 regions, which was used in the Human Microbiome Project (personal communication). As such, comparisons between studies that used different primers are difficult to make. Recent efforts by the Earth Microbiome Project (EMP) have resulted in the generation of primer sets that detect the highest diversity and are currently being used to elucidate the microbiomes of numerous organisms and environments on planet Earth. Consistent use of the same primer set across studies, particularly the use of EMP primers, will allow us to accurately compare microbiome compositions across species (as well as time and locations), conduct broad scale phylogenetic studies to investigate the evolution of symbiosis and draw more meaningful conclusions. In addition, it may help us to more readily solve the issues faced regarding unclassified bacterial sequences that may constitute a large portion of an organism’s microbiome [106] or may be responsible for differences observed [91]. As it is also easy to implement, requiring only a change in amplicon library construction with no effect on bioinformatic or computational analyses, this minor change in laboratory protocols may significantly benefit the field of octocoral microbiome research and microbial ecology in general.

New and emerging methods in microbiology are also becoming available (described in detail in [97]) and will allow a better understanding of the localization and potential functions of bacterial symbionts. Using these methods, it will be possible to highlight the different holobiont compartments where microbial processes are taking place and the mechanisms which mediate these processes. Briefly, such techniques include halogen in situ hybridization secondary ion mass spectrometry (HISH-SIMS) [236], to precisely locate microbes within-host tissues. Metagenome, whole genome and single cell genomic sequencing [237] and RNA-Seq on isolated single cells [238] will be useful to shed light on the potential functional role and life cycle of bacterial symbionts. Pulse chase isotope labelling coupled with Nanoscale SIMS (NanoSIMS), can be used to image and quantify the transfer of specific metabolites from microbial symbionts to host cells [239]. Finally, molecules within a given cell or tissue can be identified by high resolution mass spectrometry techniques, such as time-of-flight SIMS (TOF-SIMS) [240].

Once the functions of coral-associated microbes have been established, it will be important to assess how environmental and anthropogenic stressors affect the host-microbe symbioses and eventually promote microbial disease development. The goal is to better understand how microbes are related to coral health and to enable accurate predictions of resilience and responses of corals to climate change perturbations. We can then use this knowledge to identify microbes that may provide a coral with enhanced resistance to environmental stress, which may ultimately allow us to engineer the coral-associated microbiota to culture stress-tolerant corals for coral reef restoration [241, 242].

## Octocorals in holobiont research

Holobiont research has taken huge steps in recent years. Discussions on the hologenome concept have contributed significantly to this progress and identified some of the most pressing issues in this field [243]. For example, does the response to selection occur at the level of the host or microbiota? Is vertical inheritance of complex microbiomes common? And is phylosymbiosis taxonomically widespread among hosts? Research on octocorals may provide new insights to answer these questions.

Phylosymbiosis has been observed in a diverse range of organisms, including insects, rodents, and hominids [244]. Evidence of phylosymbiosis is also present within the octocoral holobionts, showing a parallel between host phylogeny and its microbial community. However, the observation that there is a significant overlap in the core microbiome between various Mediterranean gorgonian species belonging to the same genera [20], as well as an incomplete phylogenetic separation of those species [138], provides a unique opportunity to study the principle of phylosymbiosis and how phylosymbiotic signals may arise in complex holobionts and potentially shed some light on the drivers of speciation and holobiont assembly.

Vertical inheritance of a microbiome may also occur in octocorals, particularly as many species are brooding (i.e., fertilization and larval development happen within the mother colony and fully developed larvae are released). While it is likely that the microbes are transferred from parent to offspring, current investigations endeavor to address this question as well as whether core microbiome members are already present within the larval tissues prior to release. Heredity of the microbiota may also explain, in part, the spatial stability of the host-microbe associations observed in octocorals. However, there is likely also a strong selection for a specific microbiota by the host and potentially some microbes (see the “Microbiome regulation” section), which would be required for such stability, especially for life in a “microbial soup,” like the ocean. Given their selection potential and microbiota stability, as well as their associations with microbes commonly found on a range of marine invertebrates, octocorals are likely good model systems to study complex marine invertebrate-microbe symbioses. Taken together, octocorals may provide a good system to not only study coral-microbe symbioses, but also address basic questions in our understanding on holobiont assembly, functioning, and ecological evolution.

## Conclusions

Since the recognition that corals are holobionts through their intricate relationships with microbial symbionts, significant research efforts have investigated the coral microbiome composition and are beginning to focus on its functional role. Currently, we know that the microbial assemblages associated with soft corals are relatively stable and that the holobiont possesses various mechanisms to regulate its composition depending on the environmental conditions. This regulatory capacity may be one of the reasons why octocorals are so successful and inhabit many marine habitats. Connecting the functional links between host and microbial symbionts and elucidating the microbiome dynamics under various conditions will be one of the main challenges. The use of novel approaches, such as metagenomics and metatranscriptomics, combined with specialized mass spectrometry techniques will help to unravel the functions of the octocoral-associated microbes and highlight their importance for host fitness and may further reveal the potential of the octocoral holobiont as a source of new natural products and drugs. Understanding octocoral microbiome dynamics and the functional roles of all microbial symbionts within the holobiont will assist the development of strategies to help build resilience in corals under environmental change.

## Abbreviations

AHL: *N*-acylhomoserine lactone
AMP: Antimicrobial peptide
BBD: Black band disease
DOPA: Dihydroxyphenylalanine
EMP: Earth Microbiome Project
HISH: Halogen in situ hybridization
ITS: Internal transcribed spacer
LSMA: Locally stable microbial associates
MFPS: Multifocal purple spots
NP: Natural product
OTU: Operational taxonomic unit
P:R ratio: Photosynthesis:respiration ratio
POX: Peroxidase
QS: Quorum sensing
RBD: Red band disease
ROS: Reactive oxygen species
SA:V ratio: Surface area:volume ratio
SIMS: Secondary ion mass spectrometry
SOD: Superoxide dismutase
TOF: Time-of-flight
